# HCN Channels and Heart Rate

**DOI:** 10.3390/molecules17044225

**Published:** 2012-04-05

**Authors:** Pietro Scicchitano, Santa Carbonara, Gabriella Ricci, Cosimo Mandurino, Manuela Locorotondo, Gabriella Bulzis, Michele Gesualdo, Annapaola Zito, Rosa Carbonara, Ilaria Dentamaro, Graziano Riccioni, Marco Matteo Ciccone

**Affiliations:** 1 Section of Cardiovascular Diseases, Department of Emergency and Organ Transplantation, University of Bari, School of Medicine, Policlinico, Bari 70124, Italy; 2 Cardiology Unit, San Camillo De Lellis Hospital, Manfredonia (FG) 71043, Italy

**Keywords:** HCN channels, I_f_-current, heart rate control, ivabradine

## Abstract

Hyperpolarization and Cyclic Nucleotide (HCN) -gated channels represent the molecular correlates of the “funny” pacemaker current (I_f_), a current activated by hyperpolarization and considered able to influence the sinus node function in generating cardiac impulses. HCN channels are a family of six transmembrane domain, single pore-loop, hyperpolarization activated, non-selective cation channels. This channel family comprises four members: HCN1-4, but there is a general agreement to consider HCN4 as the main isoform able to control heart rate. This review aims to summarize advanced insights into the structure, function and cellular regulation of HCN channels in order to better understand the role of such channels in regulating heart rate and heart function in normal and pathological conditions. Therefore, we evaluated the possible therapeutic application of the selective HCN channels blockers in heart rate control.

## 1. Introduction

The cardiac impulse originates in the sinus atrial node (SAN), located at the right atrial endocardium, between the upper and lower cava vein, and formed by highly specialized cells able to generate action potentials (APs) which start sinus rhythm.

These action potentials are fundamental in order to allow cardiac muscle cells to contract and make the heart to play its work. Within cardiac cycle length, this means that phase 4 is characterized by a slow increase in membrane potential to the threshold for triggering a new action potential. There are many currents (both inward and outward) involved in the functioning of the SAN, able to determine the profile of the final current. In particular, the three main ones are:

- An inward current, I_f_, induced by hyperpolarization;
- An inward current of calcium, I_Ca_;
- An outward current, I_k_.

## 2. The Sinoatrial Pacemaker Currents

The pacemaker current, I_f_, is essential in the beginning and control of heart rate. It starts its active action within the end of repolarization period. Described by Brown, DiFrancesco and Noble in 1979 [1], it was called “funny” because the biophysicists did not expect to find an inward sodium (Na^+^) current in the pacemaker cells after the repolarization period.

It is activated by a membrane potential more negative than −50 mV: the more negative the membrane potential becomes, the greater the intensity of the I_f_ current. Since this current is inward during phase 4 of action potential causing a slow increase in membrane potential, researchers hypothesized [1] that could be involved in the generation of spontaneous activity; therefore, I_f_ is also increased by catecholamines, suggesting the involvement of such neurotransmitters able to influence the increasing in heart rhythm. DiFrancesco *et al*. [2] demonstrated on isolated cells of rabbit atrial sinus node that adrenaline (1–5 μM) and noradrenaline (1 μM) are able to increase the I_f_ current around the voltage range of half-activation and accelerated its activation to more negative voltages Furthermore, by adopting the same cell model, DiFrancesco and Tromba [3] showed that low doses of acetylcholine (Ach) slowed the spontaneous activity of the SAN prolonging the diastolic depolarization phase, probably due to the activation of an outward potassium (K^+^) current (I_K,__ACh_), acetylcholine able to depress I_f_ event at Ach blood concentration equal to 0.03–1 μM.

I_Ca_, instead, is activated near the end of phase 4, when the membrane potential approximately reaches the value of −55 mV. Calcium ions entrance accelerates the diastolic depolarization, which gives rise to the ascending phase of the AP.

The progressive diastolic depolarization mediated by the two inward currents is opposed by the outward current I_k_. The K^+^ efflux tends to re-polarize the cell after the ascending phase of the action potential and it continues far beyond the maximum period of repolarization, although a reduction in amplitude is observed during phase 4 of the AP period.

All these ions currents and their relative channels could be target of a pharmacological treatment able to control depolarization and/or repolarization period of sino-atrial node and, consequentially, of the heart rhythm. According to the aims of the present review, the I_f_ is going to become the main source of our discussion.

## 3. The “Funny” Current

The cardiac automatism is guaranteed by the presence of the pacemaker current I_f_ during the diastolic period, able to support the progressive depolarization of diastolic potential. Using the patch-clamp technique, the pacemaker current was primitively interpreted as a pure potassium outward current found in diastolic voltages and defined I_K2_ [4,5,6].

For many years I_K2_ was considered to act during the AP plateau, undergoing a progressive decline during repolarization. It was concurrently thought that a "background" current gradually brought the membrane potential toward less negative values until the threshold for Na^+^ and calcium (Ca^2+^) channel activation that usually triggers a new action potential.

The significant similarities between Purkinje fibers I_K2_ current and sinoatrial node cells I_f_ current erased debates about the exact source of I_f_. DiFrancesco [7,8] demonstrated that the pacemaker inward current was an inward current activated during the hyperpolarization: I_K2_ of Purkinje fibers and the I_f_ current SAN were the same expression of an equal current. Since then, lots of studies have characterized more precisely the I_f_ current in the specific myocardial cells [9].

This is a “mixed” conductance of Na^+^ and K^+^ ions [10,11] current, with a very slow kinetics until −40/−50 mV. The conductance stops during the AP depolarization period, *i.e.*, at positive voltages, although repolarization, *i.e.*, transmembrane potential difference of −40/−45 mV, makes the current switch on and progressively increase. Thus, the I_f_-current results in an inward current in the late phase of repolarization, responsible for the diastolic depolarization phase and triggering of the next action potential once threshold is overcome.

The research advancies demonstrated that a moderate vagal activity was able to control the heart rate (HR) by directly acting on the I_f_ current, in contrast with previous beliefs about the central role of I_K,Ach_ [12]. Furthermore, during a normal diastolic interval only a small fraction of the maximum conductance of I_f_ (which is approximately equal to 1pS [13]), is activated, *i.e.*, about 0.3 pA/pF. This means that the net inward current required to depolarize SAN cells is very small: for this reason, I_f_ has a large functional reserve that allows for a modulatory action exerted by neurotransmitters and hormones [14].

## 4. The HCN Family: The Mediators of the If Current

Studies [15,16] identified genes codifying for the family of ion channels that conduct I_f_ current. These genes and their products are identified with the acronym HCN (Hyperpolarization activated-Cyclic Nucleotide)-gated channels.

HCN channels are tetramers, similar to voltage-gated potassium channels (Kv) and those modulated by cyclic nucleotides (CNG). Four different isoforms (HCN 1–4) are known, all differently expressed in the heart. In particular, the HCN4 isoform is the most transcribed in the sinoatrial node, followed by HCN1 and HCN2, while HCN2 is the main transcript in ventricle [16,17,18,19,20]. In human heart, HCN4 proteins are strongly expressed in the sino-atrial node cells but none could be identified in the surrounding atrial tissue. “Funny” channels are also expressed in the atrial-ventricle node (AVN) and His-Purkinje system.

Each isoform is composed by six trans-membrane domains (S1–S6); S4 is the voltage sensor; in the pore region, between S5 and S6, a GYG sequence typical of K^+^ permeable channels could be detected; a linking site for cyclic adenosine monophosphate (cAMP) in the C-terminal region of the peptide is present and this is the key feature that distinguishes the HCN channel from other types of voltage-gated channels.

The channel is modulated by cAMP through a direct action on the channel itself, not by phosphorylations, as usually happens with other types of ionic channels [21]. The cAMP binding to HCN channels induces a conformational change of the protein: this increases the probability that the channel is open during hyperpolarization [22]. This allosteric link during the opening status of the channel is useful in stabilizing the opening conformation [23].

The importance of cAMP role in regulation of HCN channels could be found considering the inner characteristic of such proteins in controlling heart rate according to different human clinical and physiological conditions. In fact, intracellular levels of cAMP increase in the SAN cells after a beta-adrenergic stimulation, while they decrease during vagal activation, *i.e.*, when acetylcholine links to muscarinic receptors. These actions mediate the role of the autonomic nervous system on the control of the heart rate [21] and the advantages coming from the regulation of this process and, conversely, of the heart rate, independently from the myocardial contractile function.

Therefore, literature data located HCN channels in the cellular membranes, showing their presence in microdomains like lipid raft/caveolae, fundamental for a correct function of the channels themselves [24]. Combining immunocytochemistry studies, co-immunoprecipitation, Western blot and patch-clamp, the importance of the HCN4 channels localization in caveolae in the modulation of the channels has been evaluated, through the activation of specific beta-1 and beta-2 adrenergic receptors [25].

## 5. Genetic Mutations of the HCN Channels and Cardiac Arrhythmias

HCN channels’ main function is the generation of the sinus rhythm and, therefore, heart rate control. It should be expected that dysfunction in funny channels would cause arrhythmic behaviour. Molecular approaches have tried to clarify the relationships between mutations of the HCN channels genes and alterations in the cardiac function, above all according to a simple arrhythmic level.

A missense mutation (S672R) on the exon 7 in hHCN4 gene induces the production of an isoform of the pacemaker channel changed at cAMP binding site level: this was responsible for a familial form of an asymptomatic bradycardia [26].

Recent studies have underlined the existence of four different mutations associated with different types of arrhythmia in the human isoform of the HCN4 channel [27]. A mutation in the exon 5 of the HCN4 gene is functionally related to a truncated protein, unable to bind cAMP and, therefore, presenting a dominant negative effect on channel function.

Missense mutations of the same gene have been found in a Japanese family suffering from sick sinus syndrome, recurrent syncopes, severe bradycardia (39 bpm), long QT interval and polymorphic ventricular tachycardia [26,28,29,30].

Considering the complexity of the cardiac phenotype of this individuals, it was difficult to understand what was the specific phenotypic aspect associated with the mutation. Further studies are needed in order to detect the exact role of HCN mutations in developing cardiac arrhythmias.

## 6. Biological Pacemakers

Our comprehension of the molecular mechanisms of the cardiac rhythm genesis has led us to think about new therapeutic applications within the cardiac rhythm diseases field. Projects aimed, for example, at creating a cellular substrate, able to autonomously generate a spontaneous and repetitive electric activity and then to act as a “biological pacemaker”, with the aim of a possible use in the future, like substitutes of the electronic devices used at the moment.

Practically, the differentiation *in vitro* of stem cells (embryonal or adult) is induced towards a cardiac phenotype, pacemaker-like, during which the cells acquire ionic channels and proteins, necessary to generate spontaneous action potentials [31].

Further research will aim at reproducing similar features of the mechanical devices, using a genetic or cellular therapy in order to create a sort of permanent and automatically responsive pacemaker. A limit to this development is the exact identification of the gene, its outer construction and its positioning into double layer of cell membranes. A recent review shows how the use of a sequential approach is necessary in this kind of research, to combine cellular types, cultivated *in silico*, *in vitro* and *in vivo* [32].

## 7. HCN Channels, Heart Rate and Pharmacological Interventions

The importance of f-channels in cardiac rhythm creation and control has led researchers to think about the possible use of drugs to control them. Selectively blocking them could have a therapeutic role in cases in which it is useful to slow the cardiac rhythm without altering other cardiovascular functions like ventricular contractility. Nowadays, a pharmacological approach to heart rate control is possible, thanks to specific heart rate-reducing drugs. Heart rate reduction is a recognized therapeutic target in several cardiac conditions, such as ischemic heart disease and heart failure.

In patients with a coronary artery disease (CAD) a high heart rate increases myocardial consumption of oxygen and reduces diastolic coronary flow time: this promotes myocardial ischemia development. Furthermore, a high heart rate seems to induce endothelial dysfunction (an early marker of atherogenesis), probably through an augmentation of vessels wall stress [33,34].

I_f_ current is one of the determinants of the pacemaker activity; its complete blockage can reduce up to 20–30% the heart rate (heart rhythm subjects to a fine regulation from other ionic currents than I_f_, for this reason we can reach only a 20–30% heart rate reduction with I_f_-current blockers). 

Widely used drugs, like calcium antagonists and beta blockers, are not selective SAN blockers, and are even able to act in other cardiac and extracardiac districts. Nevertheless, researchers have managed to synthesize new molecules, defined as “*heart rate-lowering agents*” [35,36] and success within the field had been reached thanking of the new advances in our pharmacological understanding of f-channels [37,38].

The precursor of heart rate-lowering agents is *alinidine*, derived from clonidine and characterized by analgesic and heart rate-lowering properties and selectivity for f-channels, as is well-established by the literature data [39,40,41,42]. Nevertheless, its analogues were not able to overcome the several side effects of such molecules.

Analogues of verapamil (*i.e.*, *falipamil*, *zatebradine*, *cilobradine*) succeeded in controlling heart rates better than the former ones [43]. Zatebradine, the first deeply studied molecule of such a family, differs from falipamil in its seven atom lactam ring which increases its heart rate-lowering activity. The presence of a basic nitrogen atom, moreover, seems fundamental for the activity of this compound because the protonated form penetrates into the open pore binding to the receptor site [44].

*Ivabradine* has been obtained from the stiffening of the amine portion of zatebradine. By inhibiting the channels, it selectively reduces cardiac rhythm without influencing inotropism and avoiding the side effects of less-specific heart rate-lowering agents, such as beta-blockers and calcium antagonists.

The channel blockage caused by ivabradine reduce the conductance without modifying the biophysical properties of the pore and the nearby structures, like the voltage dependent ones [45]. Ivabradine, moreover, has a typical use and rate-dependence action. This behaviour is typical of a drug designed for a channel modulation and makes the action of ivabradine dependent on biophysical state (open-close) of the channel itself [46]. Such a peculiarity is really important: the increased pharmacological effect at higher heart rates is the main aim of the drug action because during such a condition there is an augmentation in cardiac stress caused by a compulsory need of oxygen by tissue cells. Ivabradine is unable to modify other ionic currents and it is the only inhibitor of the I_f_-current which has undergone extensive clinical development.

Its anti-anginal effect has been evaluated in different controlled clinical studies. The REDUCTION study was a multicentric one that involved 4,954 patients with stable angina pectoris. After a 4 months treatment period with ivabradine, heart rate was reduced 12.4 bpm on average [from 82.9 to 70.4 bpm, p < 0.0001], reducing angina pectoris attacks [from 2.4 to 0.4 a week]. The consumption of short-acting nitrates has been reduced from 3.3 to 0.6 units a week (p < 0.0001). The most common side effects have been: nausea (0.22%) and dizziness (0.18%), although this study showed that ivabradine is highly effective and well tolerated in the treatment of patients with a symptomatic coronary disease [47].

The BEAUTIFUL study [48] has been the first big multicentric study aiming at verifying if the reduction of the heart rate with ivabradine was linked with reduction of cardiovascular mortality in patients with coronary artery disease and left ventricular systolic dysfunction. A total of 12,473 patients, coming from 781 centres of 33 states have been enrolled. Ivabradine has been administered in 5,479 patients whilst 5,438 have received placebo. The primary endpoint was cardiovascular mortality, hospitalization for acute myocardial infarction and exacerbation or new onset heart failure. Secondary endpoints were: hospitalization for fatal and nonfatal myocardial infarction and coronary revascularization. Results pointed out that ivabradine did not affect the primary endpoint (hazard ratio 1.00, 95% CI 0.91–1.1, p = 0.94). On the other hand, considering a subset of patients with a heart rate >70 bpm, ivabradine did not lead to the reduction of the primary outcome (hazard ratio 0.91, 95% CI 0.81–1.04, p = 0.17) but resulted in a reduction of secondary endpoints: admission to hospital for fatal and nonfatal myocardial infarction (0.64, 95% CI 0.49–0.84, p = 0.001) and coronary revascularization (0.70, 95% CI 0.52–0.93, p = 0.016).

Therefore, it has been prospectively and indirectly observed [48,49] that the resting heart rate is a prognostic factor for cardiovascular events. Therefore, in a population that simultaneously had stable coronary artery disease and ventricular dysfunction (asymptomatic and/or mildly symptomatic), ivabradine was not able to reduce the combined endpoint but only the ischemic endpoints in patients with resting HR ≥ 70 bpm. Probably the basis of the partial failure of the BEAUTIFUL study has been due to the intention to consider two distinct pathophysiological conditions such as CAD and heart failure simultaneously.

The SHIFT study (Systolic Heart Failure treatment with the I_f_ inhibitor ivabradine) is an attempt to solve the problems of the BEAUTIFUL study design. It was focused on patients with left ventricular systolic dysfunction, regardless of etiology [50]. The study enrolled 6,500 patients from 37 countries, who were randomly assigned to ivabradine or placebo in addition to standard therapy for heart failure. The aim was to verify if the heart rate at rest increased the risk of heart failure recurrences. The primary composite endpoint was represented by cardiovascular mortality and hospitalization for heart failure. The analysis included patients with higher pretreatment HRs (greater than 87 bpm) and lowest ones (70–72 bpm) and demonstrated that heart rate is not only a risk marker but also a risk factor for subsequent cardiovascular events in patients with chronic heart failure. Furthermore, patients with the highest HRs in the placebo group had a primary composite endpoint incidence risk 2-fold higher than patients with the lowest HRs (p < 0.0001). The risk of events of primary composite endpoint increased 3% every rise in HR from baseline and 16% every increase of HR of more than 5 beats per minute.

Furthermore, at the 28th day, the treated patients who had a HR lower than 60 bpm showed a lesser primary composite endpoint events rate than their higher HR counterparts (17.4% *vs*. 32.4%). The reduction of the risk with ivabradine, compared with the placebo, was due to the reduction of the HR. The analysis confirmed that a high HR is a risk factor in the heart failure and that the selective reduction of the heart rate with ivabradine improves the cardiovascular outcomes.

Considering the clinical evidence currently available in the chronic heart failure field, caused by left systolic ventricular dysfunction, the utility of ivabradine seems to be limited to a restricted group of patients with a sinusal rhythm like those who don’t tolerate beta-blockers and with a heart rate ≥70 bpm, or patients intolerant to beta-blockers and unable to reach an effective bradycardization yet, or tachycardic patients in spite of a beta-block at high dosages. In experimental models, ivabradine was able to improve the ventricular function [51], in addition to the endothelial function [52], the coronary flow and the extension of the myocardial infarction [53].

The selective heart rate-lowering drugs reduce the sinusal frequency, but they do not alter the contractile myocardial properties and the ventricular electro-genesis (QT interval). The effects of the block of the I_f_-current, in fact, are limited to the pacemaker function, without any influence on the sino-atrial conduction, electrical activity in other districts, contractility, vascular resistance. The expected selectivity of action of the block of the I_f_ current may represent an important therapeutic advantage.

## 8. HCN Channels and Knock-Out Mice Models: The Experimental Face of HCN Functions

HCN channels had been extensively studied in mice models [54]. These represent the preliminary studies of a more complex understanding of their role in human beings. Baruscotti *et al.* [55] recently developed a knock-out HCN mice model in order to study the influence of the HCN4 isoform in pacemaker generation and rate control. They demonstrated a severe bradycardia (about 50% respect of original rate) and atrio-ventricular block in a mouse model in which HCN4 was ablated. Furthermore, a 70% in I_f_-current was outlined in the same models with an overall reduction of the spontaneous rate of about 60%. These results confirmed the essential role of HCN4 in adult mice in controlling the impulse generation and conduction of heart impulse.

An interesting overview on HCN actions comes from a Herrmann *et al*. study [56]. They temporally deleted HCN4 isoforms in a mice model. They observed a consequent deletion in I_f_-current and the birth of arrhythmias characterized by several sinus pauses. Surprisingly, sympathetic stimulation was able to increase heart rate, suggesting the HCN4 isoform was able to stabilize cardiac rhythm rather than accelerate heart rate. The role of HCN is almost controversial. Herrmann *et al.* [56] demonstrated that HCN knock-out adult mice succeeded in surviving. Nevertheless, HCN seemed to have an important role in embryonic mice because their absence induced early death even *in utero* [57]. Naturally, several studies are needed in order to understand the full mechanism underlining the exact role of these channels in human heart.

## 9. Conclusions

This review underlines the importance of the HCN channels both under physiological conditions (*i.e.*, control in heart rate and generation of the cardiac impulse in the SAN) and in the pathological ones (*i.e.*, using specific blockers of these channels, which can reduce the heart rate, without, however, interfering with other functions like cardiac inotropism, peripheral vasculare resistances or atrioventricular and intraventricular conduction). Further results are awaited from the SHIFT study, still in progress. Ivabradine, the first drug available in Italy from 2008 is suitable for the symptomatic treatment of the stable chronic angina pectoris with sinusal rhythm in case of contraindications to beta-blockers. Moreover new studies and analysis are necessary to understand what kind of patients can benefit from targeted drugs, because the available data have not demonstrated a definitive application field for it. From the studies available, till now, its role in therapy results uncertain, above all in terms of reduction of the cardiovascular mortality and the safety in long term.
